# Supplementary material for: Nitrate Modulates Fruit Lignification by Regulating CgLAC3 Expression in Pomelo
Source: Int J Mol Sci. 2025 Apr 27;26(9):4158. doi: 10.3390/ijms26094158 (PMC12071814; doi:10.3390/ijms26094158)
Supplement: Supplementary file 1 [file ijms-26-04158-s001.zip › ijms-3595479-supplementary.pdf]

Supplementary Information

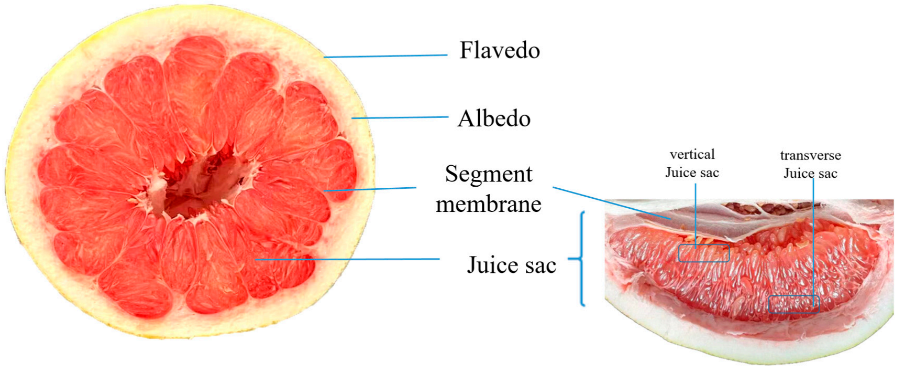

Supplemental Data Figure S1: Structure of pomelo fruits. flavedo (F), albedo (A), sement membrane (S) and juice sans which had both vertical juice sac (Jv) and transverse juice sac (Jt).

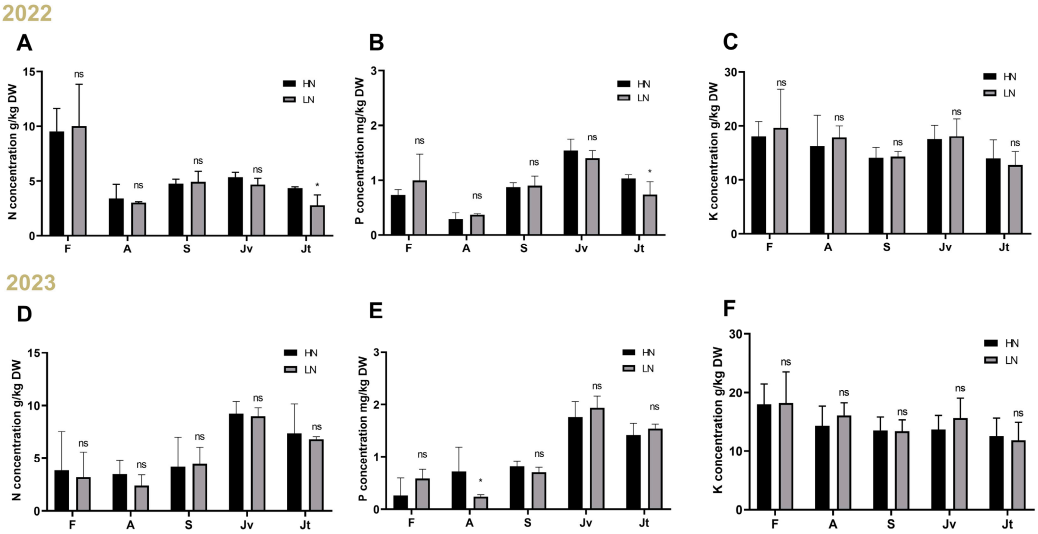

Supplemental Data Figure S2: Effects of nutrient management on N, P and K allocation in different parts of pomelo fruits

(**A**) N concentration in 2022; (**B**) P concentration in 2022; (**C**) K concentration in 2022; (**D**) N concentration in 2023; (**E**) P concentration in 2023; (**F**) K concentration in 2022. LN: application with fertilization as 160 kg N. ha^{-1}; HN: application with fertilization as 320 kg N. ha^{-1}; A-H: n =6, Student's t-test: *: $P < 0.05$. ns: no significant difference.

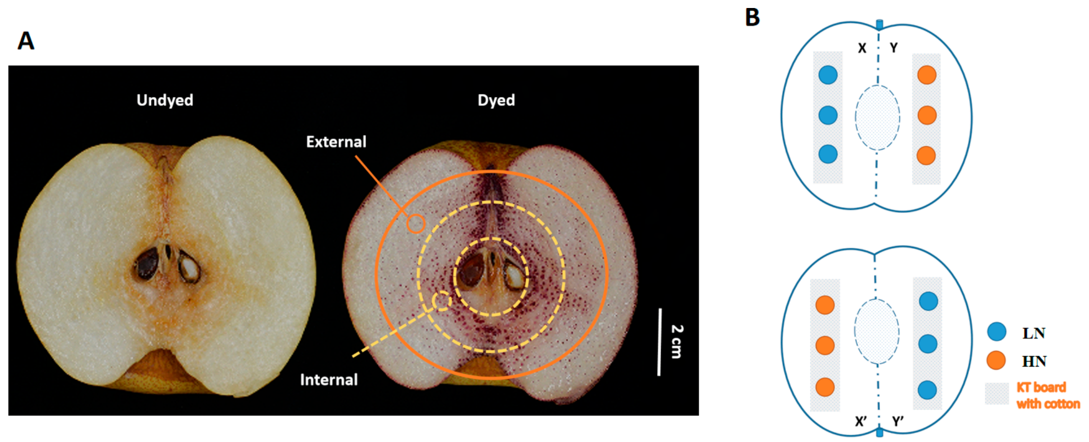

Supplemental Data Figure S3. Strucure of pear and its addition with exogenous NO_3^-

(**A**) Structure of pear fruits and *in situ* staining with Wiesner's reagent; (**B**) Diagram of adding exogenous NO_3^- on the pear fruit. .

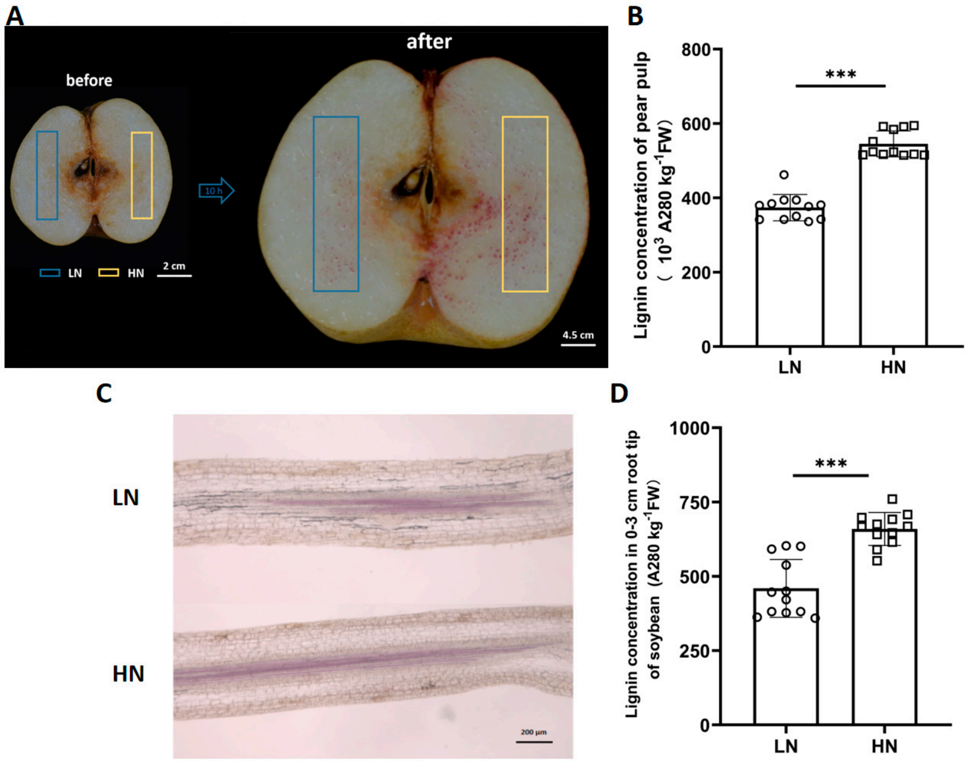

Supplemental Data Figure S4. Effects of exogenous nitrate application on lignification in pear pulp and soybean roots.

In situ staining of lignin in pear pulp (A) and soybean roots (C). Lignin concentration in pear pulp LN: 0.01, HN: 0.15 g NO_3^- kg^{-1} for pear pulp (A, B). LN: 0.20, HN: 20.00 g NO_3^- kg^{-1} for soybean roots (C, D). *n*=12, ***: *P* <0.001.

Supplementary Data Table S1. Primers for RT-qPCR analysis

	Primer Forward	Primer Reverse
CgLAC1	GGATCTTCCGCCTTGCTAGA	TGTACATTTGGTGGAAGTCAAGTC
CgLAC2	GCTATGTGGATTTTGCCGGAG	CCATGCCAATGAAGGGTGAC
CgLAC3	GCGTCTTCGCAATGGAACC	GGCTGCTTAGGACATAGCCG
CgLAC4	CTGCATCCACTTCAACGACG	GGCCAGAAAATGGGTTTGCC
CgLAC5	AACTGGCGGGTGAATGAGTA	CAAGCAACAACGAGGGAAGC
CgLAC6	CCAGCATTGGTCGAATCAGC	CCGTGCCAGTGGATTGTAAC
CgLAC7	GCCATCTTCAATCCCCAGCTT	GTAGCACTTCGGTTTCTGGC
CgLAC8	ACTCCTGGATTGTCTGCAAGG	ACAAGCATTCTTGGAGCTGA
CgLAC9	GCTTTTGCTAGCATGCGCT	CATGCCAGTGGATAGTGAGGT
GUS	GTCCTGTAGAAACCCCAACC	CTGCATCGGCGAACTGATC
EF1α	TGCAAAGGAGGCTGCTAACT	CAGCATCACCGTTCTTCAAA

Supplementary Data Table S2. RNA seq analysis

Enzyme	HN(readcount)	LN(readcount)	Log_2HN	Log_2LN	Log_2FC
PAL	3927.428057	3304.505366	11.93936913	11.69021862	0.24915051
C4H	3782.551363	2906.7875	11.88514396	11.50520989	0.379934065
4CL	712.3922856	641.3674489	9.476528084	9.325007325	0.151520759
C3H	909.7114622	917.515534	9.829265221	9.841588773	-0.012323553
HCT	1005.712141	902.4826619	9.974001714	9.817755406	0.156246309
COMT	1012.82401	883.903382	9.984167795	9.787744869	0.196422926
CSE	1013.29608	909.030013	9.984840069	9.828184118	0.156655951
F5H	94.40808749	85.37439781	6.560838549	6.415731592	0.145106956
CCR	818.983927	834.0008734	9.677691328	9.703905084	-0.026213756
CAD	335.839903	341.5996421	8.391629845	8.416162653	-0.024532809
LAC	652.7035109	451.1772653	9.350283989	8.817550562	0.532733427
PER	335.3747586	303.4685412	8.389630301	8.245403158	0.144227142
